# Challenges in Drug Discovery for Intracellular Bacteria

**DOI:** 10.3390/pathogens10091172

**Published:** 2021-09-11

**Authors:** Allison N. Tucker, Travis J. Carlson, Aurijit Sarkar

**Affiliations:** Fred Wilson School of Pharmacy, High Point University, One University Parkway, High Point, NC 27268, USA; atucker@highpoint.edu

**Keywords:** intracellular bacteria, virulence, persistence, drug discovery, infection

## Abstract

Novel drugs are needed to treat a variety of persistent diseases caused by intracellular bacterial pathogens. Virulence pathways enable many functions required for the survival of these pathogens, including invasion, nutrient acquisition, and immune evasion. Inhibition of virulence pathways is an established route for drug discovery; however, many challenges remain. Here, we propose the biggest problems that must be solved to advance the field meaningfully. While it is established that we do not yet understand the nature of chemicals capable of permeating into the bacterial cell, this problem is compounded when targeting intracellular bacteria because we are limited to only those chemicals that can permeate through both human and bacterial outer envelopes. Unfortunately, many chemicals that permeate through the outer layers of mammalian cells fail to penetrate the bacterial cytoplasm. Another challenge is the lack of publicly available information on virulence factors. It is virtually impossible to know which virulence factors are clinically relevant and have broad cross-species and cross-strain distribution. In other words, we have yet to identify the best drug targets. Yes, standard genomics databases have much of the information necessary for short-term studies, but the connections with patient outcomes are yet to be established. Without comprehensive data on matters such as these, it is difficult to devise broad-spectrum, effective anti-virulence agents. Furthermore, anti-virulence drug discovery is hindered by the current state of technologies available for experimental investigation. Antimicrobial drug discovery was greatly advanced by the establishment and standardization of broth microdilution assays to measure the effectiveness of antimicrobials. However, the currently available models used for anti-virulence drug discovery are too broad, as they must address varied phenotypes, and too expensive to be generally adopted by many research groups. Therefore, we believe drug discovery against intracellular bacterial pathogens can be advanced significantly by overcoming the above hurdles.

## 1. Introduction

Communicable diseases continue to burden the globe [1]. The cost of infections in terms of lives and financial burden is huge [1,2,3]. Six of the bacteria identified by the World Health Organization (WHO) and the U.S. Centers for Disease Control and Prevention (CDC) as threats to human health are intracellular bacteria [4,5]. Intracellular bacteria often cause chronic or latent infections, with difficult diagnosis and treatment (Supplementary Materials, Section SI) [6]. Clinically relevant intracellular bacteria can be seen in Supplementary Table S1. Evasion of host immune response by intracellular bacteria [7] makes antibiotic therapy especially important, even in immunocompetent hosts.

Bacterial resistance is a major problem [5,8,9], and resistance is inevitable with increased use [10]. Infections caused by intracellular organisms often require longer treatment durations than non-persistent infections [11,12,13,14] with multiple antibiotics [11,12,14,15], leading to resistance [4,5]. Only a limited selection of antimicrobials (e.g., aminoglycosides, fluoroquinolones, macrolides, rifamycins, tetracyclines) are useful against intracellular pathogens (Supplementary Table S1). This limited armamentarium means that each time an antibiotic succumbs to resistance, our options for appropriate treatment reduce drastically. 

Novel, nontraditional agents (such as anti-virulence agents) are a promising alternative. It has been repeatedly demonstrated (e.g., [16,17,18,19]) that intracellular lifestyles are facilitated by virulence factors, blocking which will prove to be avenues for therapy. See Supplementary Materials, Section SII for some detailed examples. Clearly, if we can identify virulence factors that enable intracellular infections, and target them successfully, we could theoretically build an array of frontline treatments for infections. *The powerful advantage of these medications would be that they do not require bacterial cell division for an effect to take place: traditional antimicrobials (i.e., antibiotics) are only useful against bacteria undergoing continuous cell division, such as intracellular bacterial colonies (IBCs).* They would not function against a subpopulation of non-dividing bacteria, such as persisters or quiescent cells. Anti-virulence drugs would function by eliminating the pathogen’s ability to survive intracellularly, regardless of whether they were rapidly dividing or not. Yet, is it so easy?

It is well recognized that antimicrobials are hard to find because this has been the major focus of the drug discovery and development community, but the challenges we face in drug discovery against intracellular bacteria have rarely been addressed. 

## 2. Challenges in Drug Discovery against Intracellular Bacteria

Several hurdles stand in the way of advancements in antibacterial treatments: (1) we do not yet fully understand the type of chemicals most suitable to become antimicrobials (this also applies to anti-virulence agents), (2) standardized databases containing information on virulence drug targets to guide drug discovery are virtually absent, and (3) we currently lack standardized and inexpensive techniques to identify and assess virulence in intracellular bacteria. These challenges will need to be met and overcome to make anti-virulence drug discovery successful.

### 2.1. Chemical Space Is a Severe Limitation

Pathogenic outer layers are not the same as ours, and hence chemicals that permeate into their cells are different. This is why chemical libraries optimized to penetrate human cells do not serve the needs of antimicrobial drug discovery [20,21,22]. O’Shea and Moser demonstrated that current antimicrobials are extremely different from typical chemicals most likely to be found in pharmaceutical screening libraries [23], which are compliant with Lipinski’s rules-of-5 (Ro5) [24]. Very importantly, they also demonstrated that the chemicals that permeate Gram-positive bacteria were different from those that permeate Gram-negative bacteria. However, further studies have shown that many Ro5-compliant chemicals are permeators as well [25,26]. Even though recent work by multiple groups [27,28,29,30,31,32,33,34,35,36,37], including us [26], have made tremendous advances in understanding which chemicals are most likely to penetrate bacterial cells (or even which chemicals are most likely to be extruded by efflux pumps), we are still far away from accurately predicting the chemical space most likely to contain the next generation of antimicrobials [38]. This situation is further complicated by the fact that differences between bacterial species (outer membrane composition, efflux pump efficiencies, and so on) make it virtually certain that our current knowledge of bacterial outer membrane permeability is insufficient. Moreover, there is no guarantee that methods built on a particular bacterial strain will work on another strain of the same species—bacteria are notorious for being “exceptions to the rules”.

**Figure 1:**
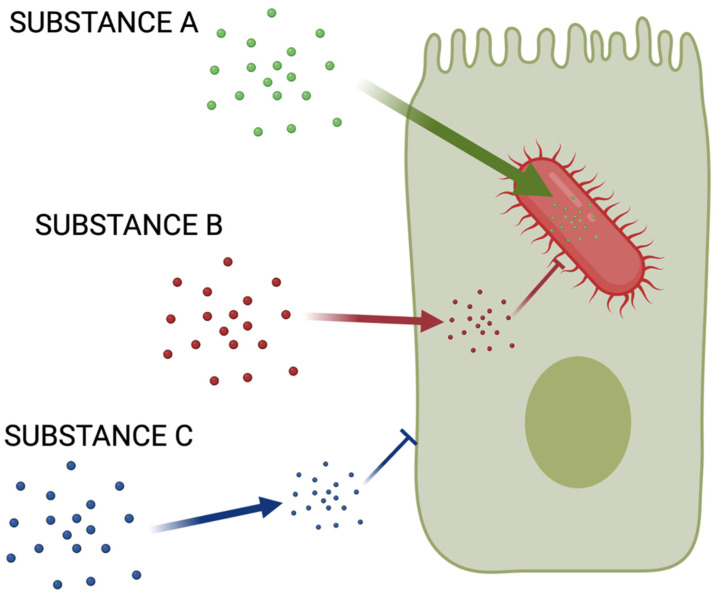
The chemical space conundrum. It is already difficult to identify chemicals that permeate bacterial outer envelopes. While recent work [27,28,29,30,31,32,33,34,35,36,37] has identified plausible characteristics enabling penetration of a few Gram-negative cells, Gram-positive permeability rules are virtually undiscovered. Additionally, only a fraction of chemicals capable of permeating the human cell can penetrate the bacterial cell to reach the cytosol. Chemicals that target intracellular bacteria must penetrate both (Substance A). Substance A must be both, Ro5-compliant, as well as bacterial cell-penetrating, which are difficult to find. Substance B, on the other hand, is useless against intracellular pathogens because it penetrates human cells but fails to reach bacterial targets. Substance C would be useless because it would not penetrate the human cell, even if it was able to reach the bacterial cytoplasm.

Now, consider that drugs that act against virulence factors of intracellular bacteria must permeate both, human *and* bacterial outer membranes. There is no escaping this fact (Figure 1). This means there are *only* two types of chemicals we can consider favorably—Ro5-compliant chemicals that easily penetrate the cytosol of mammalian cells or are facilitated to enter the cytosol through transporters. This is, perhaps, the biggest challenge we must face.

### 2.2. Finding Good Drug Targets

There are 3 different aspects of drug target selection: (1) identifying a pathway that is critical to the outcome of interest (in this case, virulence and intracellular survival), (2) identifying key enzymes of a tractable nature (i.e., enzymes whose disruption with organic chemicals is possible), and unique in the case of antimicrobials, (3) determining whether these targets are useful when attempting to design broad-spectrum agents.

*Identifying key virulence pathways.* Amongst these, our knowledge of microbial virulence factors is perhaps the most promising. We can identify genes that are directly responsible for pathogenicity or survival, as well as their mechanisms of regulation [39,40,41]. Techniques such as proteomics have helped evaluate such matters quite thoroughly (see Supplementary Materials, Section SIII for an example of application in understanding bacterial virulence). Other methods, including transcriptomics are also likely to help elucidate the basic biology dictating bacterial behavior during intracellular infections. There is no doubt that compilation of such data is ongoing, which will add tremendously to our knowledge of microbial virulence factors and their role(s) in intracellular survival. Some examples of genomics databases include the Virulence Factor Database (VFDB) [42,43] and MvirDB [44]. There are even databases to connect virulence factors to protein-protein interaction networks [45], which provide key information on the regulation of virulence as a process. While none of these databases are specifically focused on identifying drug targets in intracellular bacteria, they contain relevant data of tremendous use. Additional work will certainly provide data to aid in anti-virulence drug discovery.

At the same time, it is important to acknowledge that very little is truly understood about genetic variability for different strains within the same species. Even in the best-studied pathogens such as *E. coli* and *S. aureus*, only a handful of strains have been genotyped and made available. This is not surprising. While humans evolve over several thousand years, the time scale is literally minutes for bacteria—thus, a very large number of strains exist for each species. Enough samples to represent the genetic variability among even a single bacterial species could potentially constitute a mind-boggling amount of data. It may, however, be useful to systematically catalog strains from clinical isolates, and correlate them with patient outcomes such as clinical failure, recurrence, or even death. This may limit the number of strains for which data must be obtained as priority could be given to clinically virulent strains. Studying patients over time could also help understand how pathogens evolve within hosts to facilitate intracellular survival. 

*Chemical tractability of targets.* The next challenge is to segregate tractable drug targets from non-tractable. For the purposes of this article, we will only refer to tractability by synthetic, organic drugs because other medications such as biologics are unlikely to act directly on intracellular bacteria. Tractability by drug-like chemicals has been the subject of significant work across the past two decades [46,47,48,49,50,51,52,53,54,55]; it is also sometimes referred to as “druggability”. It is well-known that certain drug targets are much more likely to bind drug-like small molecules with high affinity [56]. The techniques are sophisticated enough to successfully identify tractable binding pockets with approximately 80%–90% accuracy, and hence can be easily used for target discovery in intracellular bacteria. Such information, when curated into a database, can provide critical clues regarding drug targets for discovery of drug targets in intracellular bacteria. However, to the best of our knowledge, only Sarkar and Brenk have used a chemical tractability method [57] to a pathogen (*P. aeruginosa* [58]).

*Broad-spectrum or narrow-spectrum?* Virulence pathways are, by definition, not critical for survival in broth—they are required only during the process of invasion and infection in a living host. These are essentially abilities developed by pathogens later in the evolutionary timeline after they encountered hosts. Thus, virulence-related pathways are not conserved across species, or even strains. For example, *S. aureus* strains have significantly varied genetic makeup, and the virulence pathways involved in infections are often different. The community-acquired methicillin-resistant *S. aureus* USA300 clone, which currently dominates skin and soft tissue infections in the U.S., does so in part because of newly acquired virulence factors lacking in the hospital-acquired *S. aureus* clones that previously dominated [59]. Therefore, it may be difficult to identify broad-spectrum anti-virulence agents. This can be countered by our knowledge of disease etiology. For example, since staphylococci are responsible for infective endocarditis in the U.S. and Europe, while streptococci are responsible for the same in other parts of the world, different narrow-spectrum agents could be used depending on local etiology [59].

**Figure 2:**
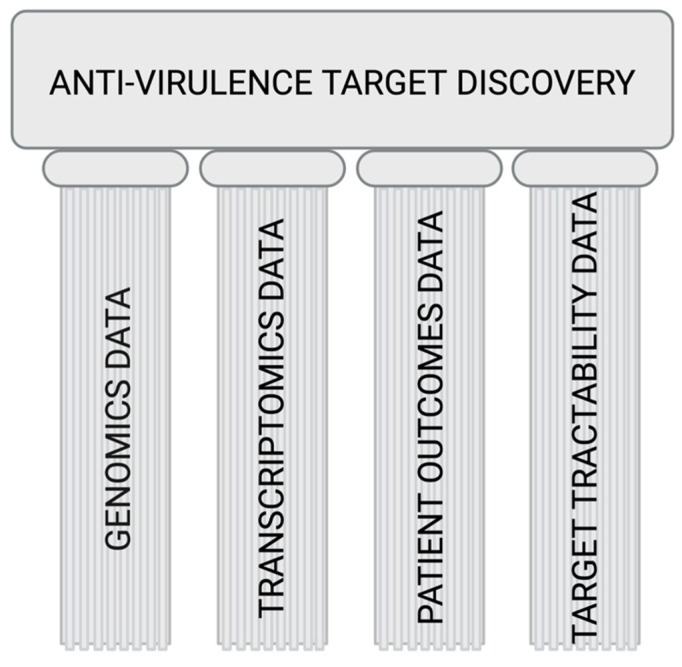
Pillars of target discovery: the essential infrastructure needed in the public domain to help facilitate and advance rational, guided efforts in anti-virulence drug discovery. The selection of targets will require an understanding of how the bacterial genome dictates virulence signaling via the transcriptome and contributes to patient outcomes. Only after understanding the chemical tractability of those targets will we be able to rationally select drug targets and focus our efforts. We envision a comprehensive platform that provides data to connect all these factors, allowing a rational approach towards target selection for the development of anti-virulence agents.

Overall, it is clear that appropriately curated and analyzed data will help us identify the best way(s) to fight intracellular infections by bringing forth good drug targets. A platform that rationally helps identify good drug targets must include, at the very least, these cogs (Figure 2).

### 2.3. Current Models of Studying Virulence

A very important factor behind the successes in antimicrobial drug discovery is the availability of broth microdilution as a phenotypic assay. Antimicrobial susceptibility measurement using broth microdilution is such a successful model that it has been standardized and used uniformly across the globe. It represents exactly what it needs to represent—the growth of the bacterium in the presence of an antimicrobial. Furthermore, reagents are inexpensive, and procedures are easy. In comparison, current models to study virulence are much more difficult and expensive [60].

Part of the problem is that there is no widely accepted, standardized method to measure virulence itself. This is mostly because virulence is an agglomerate of multiple phenotypes, unlike growth. Furthermore, standardized molecular biology techniques (e.g., RT-qPCR, Western blots, etc.) are not useful because of how virulence manifests itself—different virulence factors are often involved, and each must be measured in unique ways [40,61,62].

It is also difficult to study intracellular bacteria because the technique necessitates more expensive and challenging mammalian cell culture methods. There has been some advancement in this regard. A method has been proposed for rapid and reliable isolation of intracellular bacteria during urinary tract infections, involving rapid and inexpensive procedures [63]. However, multiple intracellular bacteria (Supplementary Table S1) manifest distinct infections of multiple tissues, requiring several highly specific models to study each infection. More generalizable methods will certainly be helpful, if they are able to adequately simulate multiple infections.

Relatively inexpensive in vitro models do exist to simulate biofilm, and they are valuable for their tractability and reproducibility. For example, U.S. CDC approved biofilm reactors are cost-effective, high-throughput methods for assessing biofilm formation; however, this method also shows attachment of the biofilm to host cell epithelia, an action not always observed in in vivo infections [64,65]. Complicating matters, antimicrobial susceptibility and bacterial growth rates may be different within and outside biofilm, and even between aggregated, surface-bound biofilm versus suspended biofilm, which can be difficult to measure in a reproducible manner. This situation is even more complicated with intracellular bacteria because it is hard to separate those surviving inside intracellular biofilms, or exiting intracellular biofilm but remaining within the cell, or perhaps even breaking out of the host cell completely. The effectiveness of an anti-virulence agent against biofilm would require accurate and precise quantitation of all these outcomes because these could lead to different clinical end points. While biofilms grown in the laboratory do not necessarily represent biofilms grown in vivo, it is always possible to follow up on the results from in vitro model with an ex vivo or in vivo model.

Animal models are ideal for studying intracellular bacteria, but they are expensive. It has always been difficult to balance factors such as inoculum dosage; high doses may be lethal while low doses result in a swift immune response [66]. Furthermore, while vertebrate models are more similar to humans, there are ethical concerns over use, and therefore must be used only when absolutely required. Invertebrate models have been introduced (e.g., *Caenorhabditis elegans* and *Drosophila melanogaster* [67,68,69,70,71]), but we are unsure if permeation of chemicals resembles human cells. Theoretically, unless similarities in permeation are well established, it is possible to find chemicals that eliminate intracellular bacteria in these models that do not reach intracellular bacteria in human cells. The major advantage of using vertebrate animals, of course, is the presence of a fully formed immune system. Just as humans, rats and mice have both innate and adaptive branches of the immune system, while invertebrates such as *C. elegans* and *D. melanogaster* do not. Invertebrates also typically do not provide large amounts of tissue for analysis [65,66]. The silkworm provides a larger invertebrate model with a simpler genome to identify genes contributing to virulence and larger tissue samples for use in virulence assays [72]. However, a simpler genome also likely means bigger differences when compared to humans.

Ex Vivo models are an alternative to animal models. Human tissues can be grown in a test tube with fewer ethical concerns, and drugs can be tested. This method permits control over experimental conditions in which the environmental aspects of bacterial cell survival and virulence can be assessed. However, even these are not cheap or easy, and the lack of an immune response coupled with a severe lack in standardization prevent large-scale expansion of this method in modelling pathogenesis [66]. Furthermore, tissue culture is not the same as humans. For example, the immune system is absent. Are these as effective as an animal model? Not likely.

In our opinion, we need new, in vitro models capable of simulating the intracellular environment. In Vitro models may never capture the complexity of a host immune response, but if we are able to simulate the stresses undergone by pathogens in an intracellular environment, we may be able to identify anti-virulence agents at the same rate as antimicrobials were discovered using broth microdilution methods. The key difference between current in vitro models and these new models should be a clear and precise demonstration of phenotypic and transcriptomic similarities. These could even be prepared separately for different cell types, but they will need to be lucid enough to run in a standardized manner, akin to antimicrobial susceptibility assays by broth microdilution.

## 3. Perspective

In the face of rising antibiotic resistance, anti-virulence therapies are a hopeful prospect in combating persistent infections caused by intracellular bacteria. From a drug discovery perspective, three changes from *status quo* are needed to truly advance the field:

1. Find better targets.
2. Develop cheaper, more effective models (maybe in vitro, for instance broth microdilution).
3. Understanding chemical space that is best to permeate both human and bacterial cells.

## Supplementary Materials

The following are available online at https://www.mdpi.com/article/10.3390/pathogens10091172/s1. Section SI: Intracellular Bacteria Are Particularly Challenging to Treat [2,3,4,5,6,7,11,12,13,14,15,73,74,75,76,77,78]; Section SII: Virulence Factors Are Important Enablers of Intracellular Pathogen Survival [7,16,17,18,19,79,80,81,82,83,84,85,86,87,88]; Section SIII: Examples of Proteomics Being Used to Understand Bacterial Virulence [89,90,91]; Table S1: A list of clinically important intracellular bacteria, including clinical conditions and therapy options [12,13,14,15,92,93,94,95,96,97].
