# Supplementary material for: Challenges in Drug Discovery for Intracellular Bacteria
Source: Pathogens. 2021 Sep 11;10(9):1172. doi: 10.3390/pathogens10091172 (PMC8468363; doi:10.3390/pathogens10091172)
Supplement: Supplementary file 1 [file pathogens-10-01172-s001.zip › pathogens-1257395-supplementary.pdf]

Section SI

Intracellular Bacteria Are Particularly Challenging to Treat

Intracellular bacteria have a parasitic relationship with their host, and in the case of obligate intracellular bacteria, cannot multiply in a cell-free environment.[1] The ability of intracellular bacteria to infect and reproduce in host cells allows them to evade the host immune system.[2] This makes appropriate antibiotic therapy especially important, even in immunocompetent hosts. In addition, their routine inability to grow in nonliving media make them difficult to identify in clinical microbiology laboratories.[1] Unfortunately, this can lead to a delayed diagnosis and, therefore, a delay in appropriate antibiotic therapy. Six of the bacteria identified by the World Health Organization (WHO) and the U.S. Centers for Disease Control and Prevention (CDC) as threats to human health are intracellular bacteria.[3, 4] *Mycobacterium tuberculosis* is of major concern. A significant risk factor for *M. tuberculosis* infections is a previous infection by the same species,[5] which suggests the pathogen persists within the host after first contact. Bacteria responsible for foodborne illness, such as *Salmonella* and *Shigella* species are also serious concerns. In the U.S., intracellular pathogens *Listeria monocytogenes*, nontyphoidal *Salmonella enterica*, and *Shigella* spp. are responsible for an estimated $2.8, $3.6, $0.1 billion, respectively, in annual healthcare costs.[6] These infections are a major burden on non-US healthcare systems as well.[7] Additionally, *Chlamydia trachomatis* and *Neisseria gonorrhoeae* cause large numbers of sexually transmitted infections globally,[8, 9] which are notorious for recurrence. Lastly, common bacteria such as *Escherichia coli* and *Staphylococcus aureus* are responsible for hundreds of thousands of hospitalizations and billions of dollars in related costs in the U.S.[10, 11] These costs are increased by the clinical complications of recurrent infections and the associated deaths. Other clinically relevant intracellular bacteria can be seen in **Supplementary Table S1**.

Persistence is a phenomenon relatively unique to intracellular bacteria.[2, 12] Persistent infections can be classified as chronic infections or latent infections.[2] Because of the bacteria's ability to persist, infections caused by these organisms often require longer treatment durations than non-persistent infections.[13-16] Furthermore, a number of these bacteria require treatment with multiple antibiotics for successful eradication,[13, 14, 16, 17] leading to resistance.[3, 4] Generally, the treatment of intracellular bacteria is limited to protein and DNA synthesis inhibitors (e.g., aminoglycosides, fluoroquinolones, macrolides, rifamycins, tetracyclines) potentially because these antibiotics may penetrate both human and bacterial cells (**Supplementary Table S1**). This limited armamentarium means that each time an antibiotic succumbs to resistance, our options for appropriate treatment reduce drastically. Thus, we must find alternatives. We propose that anti-virulence agents would be particularly useful as an alternative to antibiotics for the eradication of intracellular bacteria. However, there are technical challenges—as we will demonstrate below—that are holding back the field of anti-virulence discovery.

Supplementary Table S1. *A list of clinically important intracellular bacteria, including clinical conditions and therapy options.*

**Bacteria**	**Clinical presentation**	**Guideline recommended antibiotic therapy**	**Duration of treatment**	**Longer treatment needed?***
***Bartonella* species**	lymphadenitis<br>infective endocarditis	lymphadenitis—azithromycin<br>infective endocarditis—2-drug regimen (doxycycline, gentamicin)	Lymphadenitis—5 days [16]<br>infective endocarditis—6 weeks [16]	No

***Brucella* species**	osteomyelitis<br>arthritis	3-drug regimen (doxycycline, gentamicin, rifampin)	vertebral osteomyelitis—12 weeks [16]<br>arthritis—12 weeks [16]	Yes
Chlamydia pneumoniae	LRTI	fluoroquinolone<br>macrolide<br>tetracycline	≥ 5 days [18]	No
Chlamydia trachomatis	urethritis<br>cervicitis	azithromycin<br>doxycycline	1 day [15]<br>7 days [15]	No
Coxiella burnetii	LRTI<br>infective endocarditis [19]	doxycycline	LRTI—2 weeks [16]<br>infective endocarditis—78 weeks [16]	Yes
Escherichia coli	urinary tract infection	various	various	No
Francisella tularensis	LRTI	gentamicin<br>tobramycin	10 days [16]	No
Helicobacter pylori	peptic ulcers	3-drug regimen (amoxicillin, clarithromycin, metronidazole)	14 days[20]	No
Legionella pneumophila	LRTI	fluoroquinolone<br>macrolide<br>tetracycline	≥ 5 days [18]	No
Listeria monocytogenes	meningitis	penicillin G<br>ampicillin	≥ 21 days [15]	Yes
Mycobacterium tuberculosis	LRTI<br>meningitis	4-drug regimen (rifampin, isoniazid, pyrazinamide, ethambutol)	LRTI—26 weeks<br>meningitis—52 weeks [14]	Yes
**nontuberculous mycobacterium**	LRTI<br>osteomyelitis	3-drug regimen (azithromycin, ethambutol, and rifampin)	LRTI—52 weeks [13]<br>Osteomyelitis—26 weeks [17]	Yes
***Neisseria* species**	urethritis<br>cervicitis<br>meningitis	ceftriaxone	1 day[21]	No
**SFG *Rickettsia* species**	fever/rash	doxycycline	7 days [16]	No
**typhus group *Rickettsia* species**	fever/rash	doxycycline	5 days [16]	No
**nontyphoidal *Salmonella enterica***	infective endocarditis<br>osteomyelitis	ceftriaxone<br>ciprofloxacin	infective endocarditis—6 weeks<br>osteomyelitis—6–8 weeks	No

***Salmonella enterica* subspecies *enterica* serovar Typhi or Paratyphi**	diarrheal disease	ceftriaxone<br>ciprofloxacin	N/A	No
***Shigella* species**	diarrheal disease	azithromycin<br>ceftriaxone<br>ciprofloxacin	N/A	No
Staphylococcus aureus	infective endocarditis<br>bone and joint infection	vancomycin<br>nafcillin	infective endocarditis—6 weeks[22]<br>prosthetic joint infection—12 weeks[23]	Yes
Tropheryma whipplei	infective endocarditis [19]	doxycycline	52 weeks [16]	Yes
Yersinia pestis	lymphadenitis<br>LRTI<br>bloodstream infection	gentamicin	Lymphadenitis—10 days [16]<br>LRTI—10 days [16]	Yes

**Longer duration of treatment needed when compared to the same infection caused by other bacterial species, which are not known for intracellular existence.*

N/A: Not available from the Infectious Diseases Society of America (IDSA) or The Sanford Guide to Antimicrobial Therapy, which are common resources used to determine the clinical treatment of infectious diseases.

Section SII

Virulence Factors Are Important Enablers of Intracellular Pathogen Survival

Bacterial virulence mechanisms facilitate intracellular survival in host cells and are key to the pathogenesis of organisms known to cause persistent and/or recurrent infections. Intracellular bacteria employ two mechanisms to defend itself against immune responses: (1) evasion of recognition by the immune system and (2) modulation and suppression of the immune response.[2] These mechanisms allow bacteria to survive, move from cell to cell and cause tissue damage. Intracellular survival provides a distinct advantage by subverting human cellular envelopes to mask bacterial presence, while the cytosol provides sustenance to allow additional pathogenic processes.[24] While the exact mechanisms or processes may differ, these overarching themes are likely to be rigidly uniform across *all* species and strains of pathogens *a primis principiis*.

Avoidance of immune responses is key. Intracellular bacterial communities (IBCs) reside within the cell, undetected and unharmed by the immune system. Multiple *in vivo* models have shown that bacteria have evolved specific mechanisms to enable this. One such capability is observed in the formation of intracellular niches by uropathogenic *E.coli* (UPEC) inside host bladder epithelial cells during a urinary tract infection. These niches form through the bacterial adhesin FimH-mediated mechanism that creates biofilm-like pods inside host cells' cytoplasm. These pods help UPEC evade detection by polymorphonuclear cells such as neutrophils, and also keep out antibiotics.[25, 26] Similar intracellular niches are formed by *C. trachomatis*, where a type III secretion system (T3SS) delivers effector proteins that enable the IBC to remain undetected.[27] Importantly, these bacteria also transform into filamentous shapes while exiting these burgeoning IBCs making them too large for elimination by phagocytosis. Thus, they

can survive even upon eventual destruction of the host cell due to overcrowding. Another immune avoidance mechanism involves directly dismantling the immune-signaling cascade of the host cell. Key immune-signaling factors of the host are down-regulated by the invading *C. trachomatis*, disrupting immune responses.[28] These are critical mechanisms employed by bacteria to enable survival, and eventually, pathogenicity within the host. Blocking these mechanisms will enable a faster clearance of pathogens by the host's immune system.[2, 29]

There are specific mechanisms in place to support intracellular life, which may or may not be needed for survival and growth in broth. Pathogens use a plethora of genes to invade and infect host cells.[30-33] Often, these genes are needed to accomplish critical tasks, such as supplying key nutrients. For example, *C. trachomatis* requires lipids and acquires them by exploiting existing host pathways. Specifically, *C. trachomatis* induces fragmentation of the lipid-rich Golgi apparatus through cleavage of the protein golgin-84, which maintains Golgi matrix structure. This proteolytic action makes lipids freely available in the space surrounding the intracellular niche where *C. trachomatis* replicates. When this fragmentation process is disrupted, the intracellular growth of *C. trachomatis* is inhibited.[34] In nutrient-rich environments, bacteria still must be capable of metabolizing those nutrients efficiently. When the production of glutamine synthetase is inhibited in *M. tuberculosis*, nitrogen from the surrounding environment cannot be metabolized. This results in attenuation of the bacterial growth in human macrophages and non-virulence in a guinea pig model.[35] Similarly, a mutant of *Brucella suis* was created in which the encoding of nitric oxide reductase was disrupted genetically. This resulted in lowered rates of survival and multiplication inside murine macrophages, despite the presence of nitric oxide within the cell.[36] Host cells infected by bacteria often do not offer adequate zinc ions to permit bacterial survival. However, *S. enterica* uses virulence factors (transporters) to obtain the zinc it needs. Blocking the pathway enabling zinc uptake also blocks intracellular survival. [2, 29, 37]

Clearly, if we can identify virulence factors that enable intracellular infections, and target them successfully, we could theoretically build an array of frontline treatments for infections. The powerful advantage of these medications would be that they do not require bacterial cell division for an effect to take place: traditional antimicrobials (i.e., antibiotics) are only useful against bacterial undergoing continuous cell division, such as IBCs. They would not function against a subpopulation of non-dividing bacteria, such as persisters or quiescent cells. Anti-virulence drugs would function by eliminating the pathogen's ability to survive intracellularly, regardless of whether they were rapidly dividing or not. However, this field is brand new and full of challenges for drug discovery.

Section SIII

Examples of Proteomics Being Used to Understand Bacterial Virulence

A good example is the study of pediatric peptic ulcer disease (PUD) caused by *Helicobacter pylori*. Investigators have compared *H. pylori* strains from patients with PUD and non-ulcer dyspepsia (NUD), and through two-dimensional gel electrophoresis and mass spectrometry, identified different proteins. The proteins identified in PUD *H. pylori* strains contributed to greater mobility of flagella, increased metabolic activity (through protein flavodoxin A), and were involved in iron metabolism. In order to ensure that virulence was the determining factor for the development of PUD, impact on gastric epithelial cells between NUD and PUD was examined through an *in vitro* cell culture experiment.[38] Other such proteomics-based studies have been conducted for *Candida albicans, Candida trachomatis* and *Pseudomonas aeruginosa*.[39, 40]

Supplementary References

1. Moulder, J.W. Comparative Biology of Intracellular Parasitism. *Microbiol. Rev.* **1985**, *49*, 298–337.

2. Thakur, A.; Mikkelsen, H.; Jungersen, G. Intracellular Pathogens: Host Immunity and Microbial Persistence Strategies. *J. Immunol. Res.* **2019**, *2019*, 1356540.
3. World Health Organization. 2019 Antibacterial Agents in Clinical Development: An Analysis of the Antibacterial Clinical Development Pipeline. Available online: https://www.who.int/publications/i/item/9789240000193 (accessed on).
4. Centers for Disease Control and Prevention. Antibiotic Resistance Threats in the United States. Available online: https://www.cdc.gov/drugresistance/biggest-threats.html (accessed on 22 January 2021).
5. Millet, J.P.; Orcau, A.; de Olalla, P.G.; Casals, M.; Rius, C.; Cayla, J.A. Tuberculosis Recurrence and Its Associated Risk Factors among Successfully Treated Patients. *J. Epidemiol. Community Health* **2009**, *63*, 799–804.
6. Hoffman, Sandra, Bryan Maculloch, and Michael Batz. "Economic Burden of Major Foodborne Illnesses Acquired in the United States, Eib-140." edited by Economic Research Service U.S. Department of Agriculture, 2015.
7. 2007-2015, Foodborne Disease Burden Epidemiology Reference Group. *W.H.O. Estimates of the Global Burden of Foodborne Diseases*: World Health Organization, 2015
8. Organization, World Health. *Global Incidence and Prevalence of Selected Curable Sexually Transmitted Infections-2008*: World Health Organization, 2012
9. Rowley, J.; Hoorn, S.V.; Korenromp, E.; Low, N.; Unemo, M.; Abu-Raddad, L.J.; Chico, R.M.; Smolak, A.; Newman, L.; Gottlieb, S.; et al. Chlamydia, Gonorrhoea, Trichomoniasis and Syphilis: Global Prevalence and Incidence Estimates, 2016. *Bull. World Health Organ.* **2019**, *97*, 548–562P.
10. Simmering, Jacob E., Fan Tang, Joseph E. Cavanaugh, Linnea A. Polgreen, and Philip M. Polgreen. "The Increase in Hospitalizations for Urinary Tract Infections and the Associated Costs in the United States, 1998-2011." *Open forum infectious diseases* 4, no. 1 (2017): ofw281-ofw81.
11. Klein, E. Y., W. Jiang, N. Mojica, K. K. Tseng, R. McNeill, S. E. Cosgrove, and T. M. Perl. "National Costs Associated with Methicillin-Susceptible and Methicillin-Resistant Staphylococcus Aureus Hospitalizations in the United States, 2010-2014." *Clin Infect Dis* 68, no. 1 (2019): 22-28
12. Fisher, Robert A., Bridget Gollan, and Sophie Helaine. "Persistent Bacterial Infections and Persister Cells." *Nature Reviews Microbiology* 15 (2017): 453+
13. Daley, C.L.; Iaccarino, J.M.; Lange, C.; Cambau, E.; Wallace, R.J.; Andrejak, C.; Bottger, E.C.; Brozek, J.; Griffith, D.E.; Guglielmetti, L.; et al. Treatment of Nontuberculous Mycobacterial Pulmonary Disease: An Official Ats/Ers/Escmid/Idsa Clinical Practice Guideline: Executive Summary. *Clin. Infect. Dis.* **2020**, *71*, e1–e36.
14. Nahid, P.; Dorman, S.E.; Alipanah, N.; Barry, P.M.; Brozek, J.L.; Cattamanchi, A.; Chaisson, L.H.; Chaisson, R.E.; Daley, C.L.; Grzemska, M.; et al. Official American Thoracic Society/Centers for Disease Control and Prevention/Infectious Diseases Society of America Clinical Practice Guidelines: Treatment of Drug-Susceptible Tuberculosis. *Clin. Infect. Dis.* **2016**, *63*, e147–e195.
15. Tunkel, A.R.; Hartman, B.J.; Kaplan, S.L.; Kaufman, B.A.; Roos, K.L.; Scheld, W.M.; Whitley, R.J. Practice Guidelines for the Management of Bacterial Meningitis. *Clin. Infect. Dis.* **2004**, *39*, 1267–1284.
16. Author, The Sanford Guide to Antimicrobial Therap*y* 2020, 50 ed.; Antimicrobial therapy: city, country, 2020.
17. Griffith, D.E.; Aksamit, T.; Brown-Elliott, B.A.; Catanzaro, A.; Daley, C.; Gordin, F.; Holland, S.M.; Horsburgh, R.; Huitt, G.; Iademarco, M.F.; et al. An Official Ats/Idsa Statement: Diagnosis, Treatment, and Prevention of Nontuberculous Mycobacterial Diseases. *Am. J. Respir. Crit. Care Med.* **2007**, *175*, 367–416.
18. Metlay, J. P., G. W. Waterer, A. C. Long, A. Anzueto, J. Brozek, K. Crothers, L. A. Cooley, N. C. Dean, M. J. Fine, S. A. Flanders, M. R. Griffin, M. L. Metersky, D. M. Musher, M. I. Restrepo, and C. G. Whitney. "Diagnosis and Treatment of Adults with Community-Acquired Pneumonia. An Official Clinical Practice Guideline of the American Thoracic Society and Infectious Diseases Society of America." *Am J Respir Crit Care Med* 200, no. 7 (2019): e45-e67.

19. Fournier, P. E., F. Thuny, H. Richet, H. Lepidi, J. P. Casalta, J. P. Arzouni, M. Maurin, M. Celard, J. L. Mainardi, T. Caus, F. Collart, G. Habib, and D. Raoult. "Comprehensive Diagnostic Strategy for Blood Culture-Negative Endocarditis: A Prospective Study of 819 New Cases." *Clin Infect Dis* 51, no. 2 (2010): 131-40.
20. Chey, W. D., G. I. Leontiadis, C. W. Howden, and S. F. Moss. "Acg Clinical Guideline: Treatment of Helicobacter Pylori Infection." *Am J Gastroenterol* 112, no. 2 (2017): 212-39.
21. St Cyr, S., L. Barbee, K. A. Workowski, L. H. Bachmann, C. Pham, K. Schlanger, E. Torrone, H. Weinstock, E. N. Kersh, and P. Thorpe. "Update to Cdc's Treatment Guidelines for Gonococcal Infection, 2020." *MMWR Morb Mortal Wkly Rep* 69, no. 50 (2020): 1911-16.
22. Baddour, L. M., W. R. Wilson, A. S. Bayer, V. G. Fowler, Jr., I. M. Tleyjeh, M. J. Rybak, B. Barsic, P. B. Lockhart, M. H. Gewitz, M. E. Levison, A. F. Bolger, J. M. Steckelberg, R. S. Baltimore, A. M. Fink, P. O'Gara, K. A. Taubert, Endocarditis American Heart Association Committee on Rheumatic Fever, Council on Clinical Cardiology Council on Cardiovascular Surgery Kawasaki Disease of the Council on Cardiovascular Disease in the Young, Anesthesia, and Council Stroke. "Infective Endocarditis in Adults: Diagnosis, Antimicrobial Therapy, and Management of Complications: A Scientific Statement for Healthcare Professionals from the American Heart Association." *Circulation* 132, no. 15 (2015): 1435-86.
23. Osmon, D. R., E. F. Berbari, A. R. Berendt, D. Lew, W. Zimmerli, J. M. Steckelberg, N. Rao, A. Hanssen, W. R. Wilson, and America Infectious Diseases Society of. "Diagnosis and Management of Prosthetic Joint Infection: Clinical Practice Guidelines by the Infectious Diseases Society of America." *Clin Infect Dis* 56, no. 1 (2013): e1-e25.
24. Ray, K., B. Marteyn, P. J. Sansonetti, and C. M. Tang. "Life on the Inside: The Intracellular Lifestyle of Cytosolic Bacteria." *Nat Rev Microbiol* 7, no. 5 (2009): 333-40.
25. Anderson, G.G.; Palermo, J.J.; Schilling, J.D.; Roth, R.; Heuser, J.; Hultgren, S.J. Intracellular Bacterial Biofilm-Like Pods in Urinary Tract Infections. *Science* **2003**, *301*, 105–107.
26. Justice, S.S.; Hung, C.; Theriot, J.A.; Fletcher, D.A.; Anderson, G.G.; Footer, M.J.; Hultgren, S.J. Differentiation and Developmental Pathways of Uropathogenic Escherichia Coli in Urinary Tract Pathogenesis. *Proc. Natl. Acad. Sci. USA* **2004**, *101*, 1333–1338.
27. Chen, Y.S.; Bastidas, R.J.; Saka, H.A.; Carpenter, V.K.; Richards, K.L.; Plano, G.V.; Valdivia, R.H. The Chlamydia Trachomatis Type Iii Secretion Chaperone Slc1 Engages Multiple Early Effectors, Including Tepp, a Tyrosine-Phosphorylated Protein Required for the Recruitment of Crki-Ii to Nascent Inclusions and Innate Immune Signaling. *PLoS Pathog.* **2014**, *10*, e1003954.
28. Kawana, K.; Quayle, A.J.; Ficarra, M.; Ibana, J.A.; Shen, L.; Kawana, Y.; Yang, H.; Marrero, L.; Yavagal, S.; Greene, S.J.; et al. Cd1d Degradation in Chlamydia Trachomatis-Infected Epithelial Cells Is the Result of Both Cellular and Chlamydial Proteasomal Activity. *J. Biol. Chem.* **2007**, *282*, 7368–7375.
29. Chai, Q., L. Wang, C. H. Liu, and B. Ge. "New Insights into the Evasion of Host Innate Immunity by Mycobacterium Tuberculosis." *Cell Mol Immunol* 17, no. 9 (2020): 901-13.
30. Robino, Luciana, Paola Scavone, Lucia Araujo, Gabriela Algorta, Pablo Zunino, María Catalina Pírez, and Rafael Vignoli. "Intracellular Bacteria in the Pathogenesis of Escherichia Coli Urinary Tract Infection in Children." *Clinical infectious diseases: an official publication of the Infectious Diseases Society of America* 59, no. 11 (2014): e158-e64.
31. Freitag, N. E., G. C. Port, and M. D. Miner. "Listeria Monocytogenes - from Saprophyte to Intracellular Pathogen." *Nat Rev Microbiol* 7, no. 9 (2009): 623-8.
32. Nagamatsu, K., T. J. Hannan, R. L. Guest, M. Kostakioti, M. Hadjifrangiskou, J. Binkley, K. Dodson, T. L. Raivio, and S. J. Hultgren. "Dysregulation of Escherichia Coli A-Hemolysin Expression Alters the Course of Acute and Persistent Urinary Tract Infection." *Proc Natl Acad Sci U S A* 112, no. 8 (2015): E871-80.

33. Carlson, J. H., W. M. Whitmire, D. D. Crane, L. Wicke, K. Virtaneva, D. E. Sturdevant, J. J. Kupko, 3rd, S. F. Porcella, N. Martinez-Orengo, R. A. Heinzen, L. Kari, and H. D. Caldwell. "The Chlamydia Trachomatis Plasmid Is a Transcriptional Regulator of Chromosomal Genes and a Virulence Factor." *Infect Immun* 76, no. 6 (2008): 2273-83.
34. Heuer, Dagmar, Anette Rejman Lipinski, Nikolaus Machuy, Alexander Karlas, Andrea Wehrens, Frank Siedler, Volker Brinkmann, and Thomas F. Meyer. "Chlamydia Causes Fragmentation of the Golgi Compartment to Ensure Reproduction." *Nature* 457, no. 7230 (2009): 731-35.
35. Tullius, Michael V., Günter Harth, and Marcus A. Horwitz. "Glutamine Synthetase Glna1 Is Essential for Growth of Mycobacterium Tuberculosis in Human Thp-1 Macrophages and Guinea Pigs." *Infection and Immunity* 71, no. 7 (2003): 3927-36.
36. Loisel-Meyer, Séverine, Maria Pilar Jiménez de Bagüés, Eugénie Bassères, Jacques Dornand, Stephan Köhler, Jean-Pierre Liautard, and Véronique Jubier-Maurin. "Requirement of Nord for Brucella Suis Virulence in a Murine Model of in Vitro and in Vivo Infection." *Infection and Immunity* 74, no. 3 (2006): 1973-76.
37. Ammendola, S., P. Pasquali, C. Pistoia, P. Petrucci, P. Petrarca, G. Rotilio, and A. Battistoni. "High-Affinity Zn2+ Uptake System Znuabc Is Required for Bacterial Zinc Homeostasis in Intracellular Environments and Contributes to the Virulence of Salmonella Enterica." *Infect Immun* 75, no. 12 (2007): 5867-76.
38. Vitoriano, Inês, Kathy D. Saraiva-Pava, Alexandra Rocha-Gonçalves, Andrea Santos, Ana I. Lopes, Mónica Oleastro, and Mónica Roxo-Rosa. "Ulcerogenic Helicobacter Pylori Strains Isolated from Children: A Contribution to Get Insight into the Virulence of the Bacteria." *PLOS ONE* 6, no. 10 (2011): e26265-e65.
39. Mirrashidi, K. M., C. A. Elwell, E. Verschueren, J. R. Johnson, A. Frando, J. Von Dollen, O. Rosenberg, N. Gulbahce, G. Jang, T. Johnson, S. Jäger, A. M. Gopalakrishnan, J. Sherry, J. D. Dunn, A. Olive, B. Penn, M. Shales, J. S. Cox, M. N. Starnbach, I. Derre, R. Valdivia, N. J. Krogan, and J. Engel. "Global Mapping of the Inc-Human Interactome Reveals That Retromer Restricts Chlamydia Infection." *Cell Host Microbe* 18, no. 1 (2015): 109-21.
40. Purschke, Frauke Gina, Ekkehard Hiller, Iris Trick, and Steffen Rupp. "Flexible Survival Strategies of Pseudomonas Aeruginosa in Biofilms Result in Increased Fitness Compared with Candida Albicans." *Molecular & cellular proteomics: MCP* 11, no. 12 (2012): 1652-69.
